# Debates over orphan drug pricing: a meta-narrative literature review

**DOI:** 10.1186/s13023-025-03634-2

**Published:** 2025-03-07

**Authors:** Matthew S. Hanchard

**Affiliations:** iHuman, Social Research Institutes, University of Sheffield, The Wave, 2 Whitham Road, S10 2AH Sheffield, UK

**Keywords:** Medical sociology, Meta-narrative literature review, Orphan drugs, Pharmaceutical prices, Pharmaceutical studies, Regulation

## Abstract

Rare disease prevalence rates are increasing rapidly worldwide, as are the cost of orphan indication drugs used to treat them, posing significant strain on many healthcare systems. In response, a set of tensions have arisen within academic, activist, advocacy, industry, and policy circles over orphan drug pricing. Yet there has to date been no unifying review of the literature engaging critically with these tensions. Addressing this gap, the article examines the narratives in circulation around orphan pricing, the traditions and epistemic bases they draw on, and their points of contestation/coalescence. It does so through a meta-narrative literature review, finding three core narratives. One involves dispute over outlay costs for developing new orphan drugs, often drawing on normative health economics with a base in practical idealism. It argues that (bio)pharmaceutical manufacturers misuse policy incentives to profit excessively through monopoly capitalism. A second narrative draws on both empirical and normative health economics (often steeped in empiricism paired with a utilitarian standpoint). It contends that high orphan drug prices signify a healthy market and justifiably support longer-term innovation while promoting wider equity of access. A third (midway) narrative draws on the sociology of health and innovation studies alongside normative health economics and health policy studies to suggest alternative models of innovation and valuation. As a unifying meta-narrative, the review finds a sustained call for reform, centred on welfare economics and resource allocation, where current incentives and regulations are held to be insufficient. Overall, the article recommends that regulators look to alternative models of innovation steeped in social science thinking to modify reviewing appraisal, coverage, and reimbursement processes for orphan drugs. Also, that greater patient inclusion and transparency would help include a wider range of intangible social factors that rare disease patients face in accessing high priced orphan drugs.

## Introduction/Background

Rare diseases (RDs) affect 3.5-5.9% of the world population [1, 2] with prevalence rates rapidly rising [3, 4]. Their treatments, typically medicines with an orphan designation, are labelled orphan drugs (ODs) - and are increasingly expensive, placing strain on many healthcare systems. For instance, by 2010, eleven ODs held ‘annual sales totalling over $100 million [USD]. with 9% of [all ODs holding] blockbuster status (sales over $1 billion)’ [5]. Moreover the USA saw a 26-fold increase in orphan drug costs in 1998–2017 with ~ 12% annual increases since [6, 7]. Orphan drug revenues will likely constitute 20.3% of the world prescription pharmaceutical market by the end of 2024 [7], exceeding $1.1 trillion (USD) annual sales [8] - larger than the total gross domestic product (GDP) of many countries. In response, tensions have arisen in policy, academic, advocacy, and policy circles about the rising levels of rare disease (RD) prevalence, regulatory categories for ‘orphan’ designation, cost-valuation methods, and the broader implications for RD patients’ equitable access to treatment i.e., ODs. These often follow longer-standing debates over pharmaceutical pricing and regulatory approaches to underserved areas of medicine with unmet need, for example, past controversies over patients’ access to exceptionally high-priced HIV/AIDS drugs [5]. The latter remains pertinent with ‘newly launched prescription drugs [having] increased from a median of around $1400 a year (£1200; €1300) in 2008 to over $150,000 a year in 2021 [per patient]’ [9]. Amidst these long-standing tensions, questions remain unaddressed about the narratives circulating around OD pricing, their points of contestation/coalescence and evolution over time, the actors involved, and the bases underpinning their arguments.

This meta-narrative literature review identifies three narratives in literature surrounding orphan drug pricing. In one, commentators draw on critical normative health economics and notions of practical idealism to dispute industry calculation of OD development costs. They accuse pharmaceutical manufacturers of monopoly capitalism and incentive misuse to profiteer excessively. In a second narrative, industry-sympathetic health economists adopt either a utilitarian position steeped in positivism or lean towards notions of willingness-to-pay. They argue high OD prices signify a healthy functioning market, support longer-term innovation, and provide wider global equity of access. Many offer refined debate over OD costing calculations and approaches to valuation. A third narrative sees pharmaceutical/medical sociologists and normative economists propose alternative and novel approaches to appraisal, innovation, and valuation as way to work through the high OD price quandary. Synthesising these three narratives, the article below highlights growing consensus that incentives and policies around OD pricing are insufficient, with a sustained call for regulatory reform. It recommends that regulators look to alternative models of innovation steeped in social science thinking when reviewing appraisal, coverage, or reimbursement processes. Also, for increased patient inclusion and transparency to better encompass the full range of factors affecting RD patients’ ability to access high priced ODs.

## Method/Approach

This article presents a meta-narrative literature review (MNLR) on *orphan drug pricing* - a ‘semi-systematic approach [maintaining the] interpretive engagement, inductive reasoning, and cross-interrogation’ [10] of traditional/qualitative literature reviews. MNLRs identify the narratives, methods and epistemic traditions underpinning them, and storylines that connect them. They provide overview on ‘topics that have been differently conceptualised and studied by different groups of researchers’ [11]. They require reflexivity to ‘interpret, configure and arrange theories and concepts’ [12] into a meaningful argument, tracing ‘normal science’ in differing domains. They also rely on continual reflection to review findings [13], rather than forcing pre-existing schema onto literature or systematically quantifying articles.

The MNLR below discounts descriptions of valuation procedures, molecular compounds, or enactment of legislation/policy. It examines only literature engaging *critically* with orphan drug pricing. Following a wider project (see Funding Statement), this MNLR looks at the EU, UK, and USA only. It adopts RAMESES’ six MNLR ‘standards’ [11, 14]: (1) planning (2), searching (3), mapping (4), critical appraisal (5), synthesis, and (6) generation of recommendations. It also follows MNLR nomenclature, whereby: *meta-narratives* are [the] ‘unfolding “storylines” of research in a particular scientific tradition’ [14], each with one or more constituent *narrative*; Meanwhile, *research traditions* are ‘linked studies, each building on what has gone before and taking place within a coherent paradigm’ [12]; and each research tradition is underpinned by an *epistemic tradition* that grounds its claims in a particular way of knowing.

**Fig. 1:**
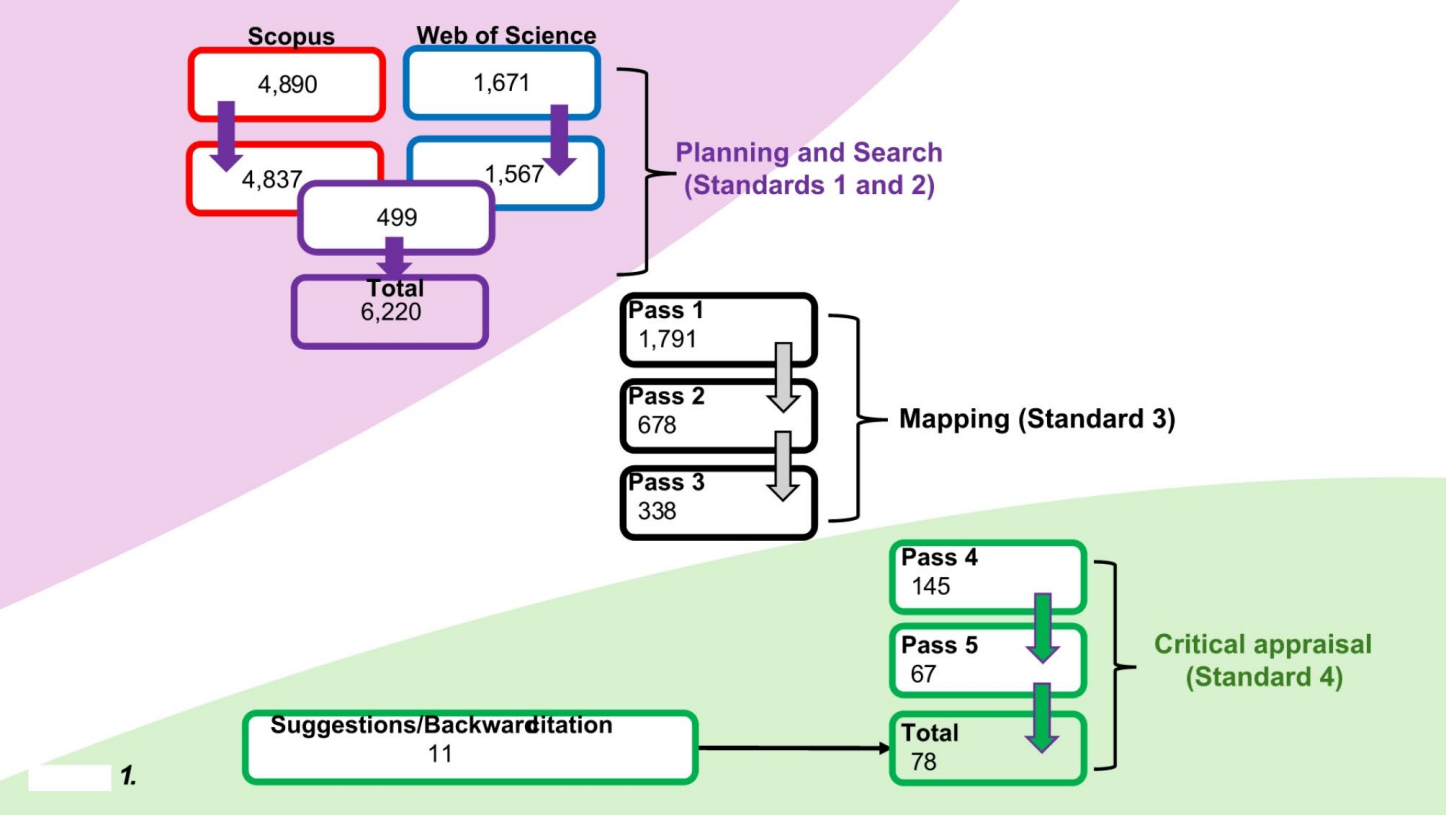
Meta-narrative literature review process

The planning, search strategy (Standards 1 and 2), and database choice were informed by three-years of ethnographic immersion in pharmaceutical debates - including eight pharmaceutical conferences, alongside various meetings, workshops, and talks, plus discussion with a subject-specific librarian, and traditional literature reviews for other publications. Clarivate’s *Web of Science* and Elsevier’s *Scopus* respectively found 1,567 and 4,828 unique items published between 1929 and 2022. A further 499 items appeared in both searches, totalling a raw set of 6,220 items after merging duplicate entries and removing erroneous ones, including two mis-indexed items dated 1929 and 1956– all other items dated 1977 or later (Fig. 1). See ‘*Availability of data and materials*’ below for a fully replicable open access search of reviewed literature items (including search terms).

Mapping the literature (Standard 3) involved categorising and winnowing it iteratively through five passes:

*Pass 1* - reading titles, outlets (journal/publisher), and abstracts (where relevant) identified a broad set of categories, whilst discounting 215 items that were irrelevant/outside scope, i.e. covering topics with similar terminology orphan products in agriculture [15]. Also, 28 that were unlocatable, i.e. journal no longer available/insufficient detail to locate and 20 that could not be accessible, i.e. URL no longer resolved/no longer in print. A further 4,166 medical studies items that held OD *pricing* as peripheral concern only were discounted, i.e. case studies on molecular entity development and/or trials outcomes. The remaining 1,791 items comprise: 977 items covering policy debate about RDs and ODs, 626 on OD pricing and valuation - including policy around access to medicines for RDs including ODs spanning bioethics, human rights, and societal values. A smaller set of 49 items cover approaches to incentivising innovation in OD development, with 136 social science items examining the logic, processes, and patient experience of orphan drug pricing. Externally, this classification resonates with other reviews of health rationing literature [16]– a field with similar concerns– which located items in either health economics, bioethics, health policy, or sociology.

*Pass 2* - re-reading abstracts and assessing journal/outlet scopes to identify research traditions, disciplinary backgrounds, approaches/methods, and narrative storylines found health policy and health economics prominent, with just 678 items to focus explicitly and critically on OD pricing debates.

*Pass 3* - excluded a further 339 items covering healthcare systems outside geographical scope, leaving 338 items to review.

*Pass 4* - re-reading abstracts, article introduction and discussion/conclusion sections, and/or full texts to exclude descriptive/explanatory items found 145 items to engage critically with high OD pricing debates.

*Pass 5*– through a full read of the remaining items, I found 67 ‘seminal’ in as far as they offered meaningful insight on the narratives [17] surrounding high OD prices Backward citation and expert suggestions increased this to the final total of 78 reviewed items, all of which have been included in the discussion below. These were extended to a final total of 108 items during the writing phase through ongoing desktop research and suggested revisions from two anonymous blind-peer reviewers.

## Narratives

Critical appraisal of the literature (Standard 4) found three narratives, respectively framing orphan drug prices negatively, positively, and in need of novel/alternative approaches to valuation. Throughout, there is a sustained call for regulatory reform– albeit framed differently by distinct positions on resource allocation and appropriate levels of state intervention.

### Narrative one: miscalculation, incentive misuse, and monopoly capitalism

In the 1990–2000 s (bio)pharmaceutical manufacturers often held R&D for new ODs at ~$800m USD [18], citing industry-funded Center for Drug Development (CDD) figures. This included ‘failures and financial charges’ [19]– with inflation calculated at 10–14%. However, in stark contrast, Prescrire International claim the actual R&D costs ran to ‘only 8 million [US dollars] (including failures, and before tax) for the 36 orphan drugs [that the FDA] approved in 1998 and 1999’ [19]. Their calculation draws on analyses from CP Tech (a project funded by grants form the Rockefeller Foundation, Ford Foundation, MacArthur Foundation, and Open Society Institute), which holds that in 1998 and 1999 ‘US private sector expenditures on clinical testing, including the costs of failures, was $283 million before taxes, and $141 million after the 50% tax credit…[while the] FDA granted marketing approval for 36 orphan products, for 39 indications… IRS tax returns [showing] US pre-tax expenditures on clinical testing, including the costs of failures [at] $7.9 million per approved orphan product (283/36), before tax, and $3.9 million after the benefits of the tax credit’ [20]– thus it suggests sizeable expenditures for bringing orphan drugs to market may sit outside clinical testing. Similarly, US Senator Metzenbaum [21] reported that in 1990–1991 R&D costs ‘for high-priced blockbuster drugs [sat] between $10 and $45 million (USD) for all but one company’ [22]. By the late-2000s, the CDD revised their calculation to $2.6b USD [23]. However, the Drugs for Neglected Diseases Initiative (DNDi) estimated €100–150 million as the true R&D cost for bringing a new OD to market [23]. Amidst these sizeable disparities, recent figures show OD R&D at around $700m USD [24] - seven times higher than DNDi’s lower-end estimate of €100m and < 26.92% of CDD’s revised calculation of $2.6b USD.

Dispute over OD R&D calculations often rest on an underlying assumption that the outlay costs for new ODs are the root cause of their high prices. The large disparity for example, can in part be attributed to differing calculations and ways of accounting for failure, where for instance, 47% of orphan drugs in development did not pass from phase II onto phase III [25], likewise the smaller market for rare disease patients mean smaller likely sales to return the offset capital cost for R&D.

However, financial reports 1999–2018 across the fifteen largest pharmaceutical companies show the R&D spend for ODs accounting for just $1.4 trillion (USD) of the total $7.7 trillion spent getting them to market [24]. Meanwhile, $2.2 trillion (USD) was spent ‘on costs relating to selling, general, and administrative activities - a category that includes marketing and advertising, as well as almost all other business costs not directly attributable to [R&D]’ [24]. However, it can be ‘difficult to [accurately] link drug prices to the receipt of public support for basic biomedical research’ [26], making any calculation of their actual costs relatively opaque. Thus, the first narrative thread sees ongoing debate over what should/should not be attributable as R&D costs, primarily steeped within empiricist health economics. By extension, a smaller sub-thread of literature holds that charitable organisations and public monies often cover or subside R&D costs for ODs [27] through upfront investment to contain costs, with payers still encountering high list prices - meaning that they effectively pay twice for the same ODs [23].

A second thread blames regulatory shortfalls for creating conditions that spawn industry-wide incentive misuse and excessive profiteering. Some authors argue the lack of requirement for regulators to conduct a health technology assessment with cost-effectiveness as criteria opens ground for monopoly-formation– with licence exclusivity on specific indications (i.e. ODs) leaving patients’ with limited alternative but to find a way to pay [28].

Others attribute it to the USA’s 2017 median cost per patient being 5.5 times higher for ODs than non-ODs [29]. Here, suggestions arise around allowing competitors ‘on the market when sales of an orphan drug reach $200 million [USD]’ [28] to encourage price competition. In-line with a free-market, however, a caveat follows that it would not mean ‘halt[ing] the sale of the first drug or [mean] tell[ing] a company what it can charge’ [28]. Others argue increased OD approvals are the issue, and ‘threaten the sustainability of healthcare systems around the world’ [30]. In part, because the (in)elasticity of healthcare markets mean that ‘[u]nlike consumers of ordinary goods, consumers of patented medicines—also known as patients with medical needs [cannot] defer consumption until prices fall’ [31]. Other authors identify ‘indication creep’ in ODs that can also be used to treat more common ailments. Eli Lilly’s ‘Prozac’ (fluoxetine), for instance, gained FDA OD designation for ‘autism and body dysmorphic disorder in children and adolescent[s], but [was later] widely administered to treat depression’ [32]. This included uninsured patients with (30-50%) 340B discounts, enabling Ely Lilly to reap exceptionally high rates of return [33]. Thus, by 2015 ‘seven of the top 10 best-selling drugs worldwide [held] an FDA-approved orphan indication’ [32]. For clarity, as a federal programme in the USA, 340B Drug Discounts enable ‘qualifying hospitals and clinics receive Medicaidlike discounts from manufacturers on qualifying outpatient drugs of around 55%’ [34]. Although 340B discounting does ‘allow covered entities to purchase discounted drugs prescribed to all their patients, including patients with Medicare or private insurance… [it] does not require covered entities to pass on cost savings’ [32]. The latter became a point of lamentation following a 2010 amendment to the Patient Protection and Affordable Care Act (PPACA), which saw 340B discounts expand to ‘children’s hospitals, free-standing cancer hospitals, critical access hospitals, rural referral centers, and sole community hospitals’ [32].

Elsewhere, the European Commission (EC) sought legislation and approvals for orphan drugs to be harmonised across member states from 1997 onwards [35], with 1975 EEC directive 75/318 as its foundation, providing ‘standards and protocols for the performance of tests and trials on proprietary medicinal products [as] an effective means of control’ [36]. It also set out a clear statement that the ‘value of data on the therapeutic efficacy and safety of a [drug]. will be very greatly enhanced if such data come from several competent investigators working independently’ [36]. The directive was later revised and extended into regulations 141/2000 [37] and 83/2001 as the first EU-wide specific orphan drug legislative framework items, with its first substantive review taking place in 2005 [38]. In response, critical debates over orphan drug prices and associated incentive misuse began slightly later in the EU than in the USA. Here, (bio)pharmaceutical manufacturers have been accused of misusing policy incentives to obtain licences for existing ODs, and then profiting from their reuse rather than developing anything new [39]. Similarly, a practice called ‘evergreening’ sees ‘minor alteration to an existing invention [generate profit from] a secondary patent…[with] 78% of new [OD] patents correspond[ing] to drugs already on the market’ [23]. Existing policy also enables (bio)pharmaceutical manufacturers to profit from new licences with minimal R&D outlay by making ‘minor chemical variations relative to a drug already on the market within a given therapeutic class’ [40] - labelled ‘me-too’ drugs. Here, in ‘1,345 [EU] new drug approvals between 2000 and 2014… 51% [were] modified versions of existing medicines…[yet] only 1% were considered a therapeutic advancement’ [23]. As an example, BioMarin’s 3,4-diaminopyridine (3,4-DAP) treatment for Lambert-Eaton and congenital myasthenic syndromes ‘[had] been produced by a small drug company on an unlicensed basis [at] between £800 (€945 EUR; $1285 USD) and £1000 per patient per year [PPPY]’ [41]. Once licensed, it was marketed and sold across the EU in slightly modified form ‘amifampridine’ and ‘Firdapse’ at ‘£40,000 to £70,000 per patient per year—a 50-fold to 70-fold increase’ [39], but with no evidence of clinical advantage. Similarly, discussion of ‘excessive stratification’ see drug manufacturers being accused of strategically seeking to increase the indications of each drug, consequently increasing both its per unit cost and sales volume. Here, commenters cite examples such as biotechnology firm Amgen’s Epogen (epoetin alfa) launched in 1989 ‘to treat anemia during the terminal phase of renal failure…’.

[Through fewer] than 78,000 patients, Epogen generated sales of $5 billion in 2001… [after] Amgem’s having the drug approved as well for high-prevalence therapeutic indications, including the recovery of red blood cells in patients suffering from bone marrow suppression caused by anti-HIV drugs or chemotherapy’ [42].

In rebuttal, others notes marketing authorisation is conducted at EU-level while deciding ‘the price of a medicine and its coverage by the health care systems (reimbursement) is a national competence of [individual] Member States’ [43]. Some blame the EC for ‘not creat[ing] an oversight body to regulate prices and protect consumers from market abuse [unlike] other state sanctioned monopolies’ [44]. As redress, they suggest a turn to anti-competition law [44] to treat incentive misuse as collusive behaviour (typically reserved for cartels’ and/or other major abuses of power), i.e. sanctioning (bio)pharmaceutical companies via Article 101 of the Treaty on the Functioning of the European Union for commanding an overly dominant or substantial part of an EU internal market which could feasibly foster unfair trading conditions. In contrast, within this narrative thread argues that a lack of regulation is not in itself to blame per se, adding that:

[It is] not actually a “free market” based on supply and demand with minimal government intervention…[but] highly manipulated, with numerous government programmes…[that] may not be focused on achieving the best prices [45].

As such, incentives like intellectual property (IP) protection and the ability to licence slightly modified versions of the same OD render (bio)pharmaceutical companies’ competitiveness questionable. Thus, this narrative portrays ‘a market with legally sanctioned exclusivity, [whereby] each company *is* the market; [with] no other participants’ [44] and where ‘IP architectures’ form layered obdurate structures limiting any form of challenge [46]– returning to an underlying contention that their high prices can be linked to the inelasticity of OD market dynamics.

A third thread continues to generate new terminology for profiteering practices but makes connection with notions of monopoly capitalism rather than blaming outlay cost calculations or regulatory shortfalls. For instance, by “artificially” subdividing diseases ‘to create small subgroups of patients that fall under the orphan drug prevalence threshold’ is often labelled ‘salami-slicing’ [23]. It means (bio)pharmaceutical companies can profit from additional licensing and exclusivity incentives for nominally different products. Similarly, when ‘[b]rand-name [ODs] are introduced with high launch prices and [then] experience high annual price increases’ [47], their artificial inflation ‘games’ the system. The latter can be connected to: ‘drug switching’ (discontinuing a cheaper drug to enforce uptake of more expensive alternative); ‘pay-to-delay’ tactics, ‘where competitors are rewarded for delaying the launch of competing products’ [45]; and/or ‘share buy-backs’ - where short-term profits are placed ahead of longer-term investments in future innovations, raising concerns over the impact on longer-term market security and sustainability. For example:

[From] 2007 to 2016, the 19 pharmaceutical companies in the S&P index in January 2017 spent $297 billion repurchasing their own shares, equivalent to 61% of combined R&D expenditures…[using] diverted R&D funds—which invest in future innovation…[and] passing on monopoly profits to today’s shareholders [23].

Moving onto a structural level, corporate acquisitions like Sanofi-Aventis’ >$20 billion (USD) purchase of biotechnology firm Genzyme are also seen as narrowing manoeuvres akin to monopoly-forming around specific therapeutic areas [48]. Such practices harbinger an industry-wide narrowing of OD R&D focus to indication/disease specialisms with high-profit yields [49]. With Genzyme, the profitability of ‘enzyme replacement therapies (ERTs) served a small patient population [but held] steep reimbursement rates’ [49]. Other companies have science clammered to ‘recreate what made Genzyme great [by] focusing exclusively on [particular] rare diseases, even at the expense of a more diversified portfolio’ [49]. Following on, first generation disease-modifying treatments (DMTs) for multiple sclerosis (MS) increased price annually 5–7 times ‘higher than prescription drug inflation [between 1993 and 2013]…from around $8,000-$11,000 USD per year to around $60,000…2 to 3 times higher than in other comparable countries’ [50]. It suggests a process of rare disease burden being increased through monopoly-forming around specific indication and therapeutic area specialism [50].

In summary, literature within the first narrative argues that: OD R&D costs are “artificially” inflated; industry misuse of regulatory incentives garners excessive profiteering; wind that unfettered monopoly capitalism around indication and therapeutic area specialisms risk further heightening unmet need. It primarily draws on normative health economics, with a base steeped in practical idealism, and involves input from academics, policymakers, and to a activists. Some commentators blame regulatory shortfalls, others blame industry– but all find that regulatory reform is needed, with a free-market model ill-suited to the inelasticity of orphan drugs.

### Narrative two: supporting healthy markets, wider access, and future innovation

A second narrative sees many claims in the first one refuted and/or refined, with high OD prices justified. It is worth noting here however, that debate largely centres around the USA, wither fewer European scholars aligning with market-based solutions. Within this, one thread holds that high OD prices signify a healthy market - that existing regulations striking the right balance with a fear that lowering costs could ‘jeopardise the continuity of supply, drive manufacturers out of the market and reduce investment’ [51]. It connects with critique of existing policy incentives, where in the USA Congress should ‘be cautious about expanding [Priority Review Voucher] PRV schemes’ in case it ‘.decreases the expected price…’; a factor that could de-incentivise development of new ODs’ [52]. From a more neoliberal position, others argue there is no entitlement to affordability of medicines developed by private companies, adding that OD price controls:

…based on the degree to which public funds contributed to its development are not just unfeasible to implement, but also a distraction from more far-reaching efforts to improve the affordability of *all* medicines. Attention should instead be focused on developing practical solutions that ensure that clinically valuable new drugs continue to be developed and are accessible by all patients in need [26].

However, framing high OD prices this way detracts from their wider impact on healthcare systems; ODs account for only ~ 0.5% of healthcare budgets, especially with existing cost-containment measures against excessive profiteering in place [3]. For example, regulators such as EMA’s Committee for Orphan Medicinal Products (COMP) are able to temper costs in Europe by reducing the market exclusivity period of an orphan drug from ten years to six [53]– under EC Regulation 141/2000 if they find a lack of significant benefit (Article 3), or if COMP find that that sufficient profit has already been made from the drugs’ sales (Article 8.2). However, the latter has only been triggered once in the last twenty years [54], indicating that ODs are held to foster and fit within a healthily functioning market when viewed as a whole.

Other commentators argue overcoming challenges inherent with OD R&D (i.e. small sample sizes for clinical trials, high failure rates, and lack of comparable research) can command ‘higher prices and/or compensating incentives’ [55]– including closer regulator-industry negotiations in initiatives like adaptive licensing [56]. Doing so could sustain innovation, support ‘equitable health systems, and [promote] a productive biopharmaceutical industry in Europe’ [56]. Regulatory change around OD pricing, they add, should be light-touch - the risk ‘is not that there will be no more orphan medicines, but there may be no more orphans launched in Europe’ [55]. However, the ‘trend is inexorably [moving] towards international reference pricing, [which means] orphan drug developers may hesitate to launch in lower-priced regions for fear of contamination of their higher price’ [55]– a factor already threatening EU-wide OD shortages [57].

In a second thread, OD prices are justified over the longer durée and lauded as a source of support for innovation. Here, it is often considered that the ‘development and use of new drugs has resulted in significant increases in longevity and health. [and that it is] highly cost-effective’ [58] in the long-run. This suggests that ODs offer good value for money on public investment, despite their high list prices. This resonates with early discussion of cell and gene therapies (CGTs), when they were first emerging, their exceptionally high prices were expected to lead towards future cost-savings across various therapeutic areas - and therefore they were held to offer excellent value for money in the long-term too. For example:

when a disease is designated as “genetic,“.it can refer to an alteration in the germ cell that is passed onto offspring…[and/or] mutations [that] take place outside the germ cells, in the cells of the body. The prevalence of the former is not nearly as great as the latter. Cancer can be “genetic” in both senses. Efforts to identify and treat genetic disease based on acquired cell mutations could have a greater cost impact, in terms of reducing resources devoted to hospitals, physician payments [59].

Elsewhere, while high OD prices are justified, overzealous regulation and lack of national protections in economic policy are blamed for access issues that foster artificially inflated consumer prices. Here, global price differentials related to intellectual property (IP) are held to at fault. For instance, following public investment subsidising Sanofi’s R&D for *Fabrazyme*, an enzyme replacement therapy for Fabry disease, ‘patients have paid monopolistic prices…[whilst] not being saved by the drug, because full doses are being exported to maintain market share in Europe, even though an alternative is available [there]’ [60]. This leads to an argument that:

“messing with patent rights” is a greater evil than promoting competition and access to drugs, especially when Europeans have such a competitive market where they enjoy full-dose treatment and alternative medication[s] [60].

Similarly, ‘[d]rug prices in the top 5 countries are almost five times as high as they are in the bottom five countries’ [58]. These price differentials are to ‘grow with the length of the market presence…[albeit] managed more aggressively by the reimbursement authorities in EU…’ [61] than the USA– highlighting a disparity between the two regulatory landscapes. Here, cost per patient per standard dose models correlated country-by-country on per capita income shows that ‘the price of [ODs] is [consistently] lower in low-income countries’ [58]. Thus, strategic price discrimination ensures equitable access to ODs albeit:

[while] profits are generally higher under price discrimination than under uniform pricing…the ability to price discriminate is more likely to increase the wellbeing of society as a whole (“social welfare”) than it is to increase consumer wellbeing [58].

The negative framing of high OD prices above can be viewed as narrowly internalist and steeped in (neo)liberalist thought, based more on notions of individual benefit than collective good. Expanding on OD cost distribution in more detail, the argument for high prices can be further bolstered through socio-demographics:

When price is defined as the amount paid by the patient, there is an inverted-U-shaped relationship between income and price…[the] lowest income category pay 25% less than high income people… but people in the middle income category (whose income is 125–200% of the poverty line) pay 6% more than high income people…[because] people in the middle of the income distribution are less likely to have prescription drug insurance than either high-income people (who have employer-based coverage) or people below the poverty line (who have Medicaid coverage) [62].

This contention aligns with a ‘squeezed middle’ rhetoric in American economics, whereby high OD costs disproportionately affect middle-income earners. Thus, it challenges the validity of PPPY cost-models being used to berate high OD prices (cf. [39]). Instead, it suggests that health economists ought to examine a wider range of social factors. Similar arguments are made in Europe too, that OD prices are justifiable on the whole, with external reference pricing (ERP) and international price comparisons generating price heterogeneity across the EU due Handfield to some member states more/less able to offer rebates than others [21]. Those with lower gross domestic products (GDPs) tend to pay relatively more for ODs than higher GDP counterparts. However, some authors contend that ERP ‘is not based on rational economic theory or evidence’ [63] and as such should only be used alongside - not in place of - other assessment methods, with market competition often far more effective at price reduction.

In a fourth thread, the valuation methods used to criticise high OD prices are challenged. For example, recent analysis of EURIPID survey data across thirty-two European countries finds the price that ‘publics pay for ODs is often lower than the published list price’ [64]. Thus, it calls into question a core point of reference (and underlying empirical base) for many of the arguments claiming ODs are priced too highly. Many of the ‘documents on price formation are [commercial and thus] classified’ [65], however, leading to uncertainty over the actual mechanism for setting OD list prices and/or their finally negotiated price. Even in accepting list prices as a base measure, some authors dispute normative health economists’ use of cost-opportunity models (cf. [66]). Doing so only ‘consider[s] values and costs in the framework of welfare economics…and utilitarianism’ [67], placing critique of high OD prices on literature at odds with the wants of a wider public who fund and pay for ODs. Instead, ‘[s]ocieties and individuals express their choices of value through their actions (so-called ‘revealed’ or ‘expressed’ preferences).[which] does not follow free market principles’ [67]. Thus, their position aligns with notions of practical idealism and willingness to pay. By comparing data on prophylaxis and on-demand treatment costs for haemophilia, for instance, Feldman et al. [67] show that reimbursement decisions are not necessarily made on a rational basis with future-cost savings in mind. Moreover, that despite their seemingly high lists price, high priced ODs often offer cost-savings when modelled around a wider range of social measures such as quality of life (i.e. QALY) and when calculated over a longer period - offering grounds for a call to reconfigure health economic valuation, and particularly those using list prices alone to berate high OD prices.

In juxtaposition, research funded by UK industry trade member charity ‘Office for Health Economics’ (OHE) argues *against* any change to the ‘Office of Fair Trading’ (OFT) ‘Pharmaceutical Price Regulation Scheme’ (PPRS) [68]. Here, QALYs are used within health technology assessments and by regulators worldwide, ‘including [the] National Institute for Health and Clinical Excellence (NICE) in the UK as well as the Institute for Clinical and Economic Review (ICER) in the US [albeit]… promot[ing] equal value Life Year Gained (evLYG) over the QALY’ [69]; notably in response to the criticism in the US that the QALY discriminates against disabled persons in situations where therapy increases life-years gained.

Where the OFT proposed to move away from a PPRS based mainly on profit control towards one based on value-based pricing– as an ‘ex ante centralised government price setting [mechanism] based on a cost-per-QALY’ [68], they argue PPRS offers ‘no mechanism for ensuring that relative prices reflect value’ [68], risking potential for unfettered price inflation. However, OHE contend instead that doing so ignores market externalities, where rewarding early innovation is likely to see costs rise with uncertainty over how to measure later benefits, or to compare first-in-class products with others [68].

Likewise, It follows that the value of an OD cannot be estimated until ‘its efficacy is demonstrated in the adjuvant setting’ [70], rendering many accusations of ODs being over-priced unfounded. Such arguments run contrary to wider social measures being used to set prices upfront. Instead, high OD prices are argued to be warranted when considering future cost-savings, i.e. that list prices alone are not enough. What combines their accounts is an underlying contention (especially prominent in the USA) that the free-market will, over time, temper the high costs of ODs.

Following on, a fifth thread revolves around national economies, where less state intervention is urged - the American healthcare system already contains costs effectively through free-market logics:

Unshackling the Centers for Medicare and Medicaid Services [CMS] from restrictions imposed by the Medicare Prescription Drug Improvement and Modernization Act of 2003, which prohibits price negotiation, would actually restore some free-market mechanisms to control costs [70].

However, this line of reasoning clashed with implemented policy in late-2010s expansion of pharmacy benefit managers (PMBs) ‘who administer drug benefits for health insurers and self-insured companies, negotiate the prices that will actually be paid, and determine how much reimbursement pharmacies will receive’ [71]. It also runs counter to the Inflation Reduction Act 2022 and associated Medicare drug price negotiation programme– both of which enable the CMS to negotiate drug prices directly with manufacturers.

In summary, literature within the second narrative follows that: high OD prices represent a healthy market; prices are artificially inflated by global market competition; differential pricing sees European and American markets subsize access elsewhere to increase broader equity of access; high costs now lead to lower future costs whilst supporting innovation; and that framing high OD prices negatively tends to lack account of wider social measures or expressed preference. Arguments tend to draw on both empirical and normative health economics traditions, albeit with a positivist bent, and include input from academics (independent and industry sponsored) and policymakers with differing positions over the relevant level of state-intervention required to contain OD prices.

### Narrative three: rethinking innovation, resource allocation, and regulation

A third narrative moves beyond the impasse of debating whether high OD prices are justified or not and instead seeks novel approaches to resource allocation, reimbursement, valuation - without harming industry, markets, or innovation cycles. Within it, one tread looks to reconcile individual and societal willingness-to-pay with the finite limits of healthcare budgets. For instance, comparing FDA novel drug approvals 2011–2015 with research costs, patient numbers, and sales shows OD R&D costs are just 23% of non-ODs [72]. It leads to suggestions of ‘…a method for establishing a reasonable price for [ODs] where a value-based price is deemed inappropriate.[for] determining the maximum allowable price society should be willing to pay’ [72]. Others compare OD prices with intrinsic costs to society if an RD is left untreated, holding the latter responsible for driving OD prices ‘to the upper limit of what health systems *can* pay’ [23]. However, ‘set[tting] aside comparative cost even after accounting for distributional and ethical constraints would be truly to act as if money is no object’ [73]. That is, payers and regulators are subject to finite staff resources and budgets too, and heavily strained - making them unlikely to implement any such new scheme [74]. By 2017, for instance, the FDA had a ‘backlog of 200 pending designation requests and [aimed to] establish procedures for vetting such requests within 90 days [74] following a four-fold increase in new OD submissions from 10% of all approvals in 1998 to 44% in 2017 [6]. In the UK, the NHS face similar issues in ‘respond[ing] to drugs being approved more quickly by licensing authorities, often on the basis of less mature clinical evidence [like real-world data]’ [75].

In less utilitarian terms, regulators’ refusal to pay (reimburse) ODs can incite ‘intense criticism from patient groups, patient associations and clinicians…[prompting] stories about families taking desperate measures to raise funds to pay for treatments’ [76]. Such evocative and highly publicised stories are often steeped within persuasive rhetorics, practical idealism, and figure-pointing; purchasers ‘accuse manufacturers [of] unethical pricing…[while] manufacturers accuse the purchasers of being unwilling to cover foregone R&D costs’ [76]. Here, political economic analyses are poised as a good way to unpack the vested interests behind these positioning games, and to better see where patients’ willingness-to-pay sits [76]. Others follow suit, highlighting unevenness in how Ds for different RDs are perceived. For example:

[with] drugs related to end-of-life care (notably in cancer), there is a belief that extensions to the last months of life are particularly valuable to patients and their families although the empirical data on public preferences for funding such drugs are equivocal [77].

A tributary debate follows-on to: on the one hand, that funding ODs means ‘costs will be borne by other, unknown patients with more common diseases who will be unable to access effective and cost-effective treatment’ [78]. On the other hand, that decision-making involves a broader range of services and considerations [79] than reported. Thus, because ‘[t]he consumer and taxpayer ultimately shoulder [OD] cost via insurance premiums and co-pays, taxes, cost-shifting, rationing, and reallocation of resources’ [80] alternative criteria are needed to justify their cover. As a proposed solution, regulators are urged to make more of equity weighting (i.e. QALY) measures around health inequality and prevalence rates to establish payment ceilings [81]. Here, implementing risk-sharing agreements between regulators and pharmaceutical manufacturers, with performance-based measures driving innovation could fairly temper costs [81]. In short a differentiated approach is put forward, with *levels of rarity* an important consideration.

Others locate unevenness (tied to costs) in access to ODs for different RDs [82], with appraisal decisions typically using cost-effectiveness analyses *before* comparison to willingness-to-pay thresholds. However, the latter draw on opportunity costs with ‘no universally agreed mechanism for setting such thresholds…[harbouring].considerable international variation’ [82] and in-built ‘bias against less treatable diseases’ [75]. As a proposed resolution, UK NICE accountability for reasonableness criteria could offer a base model for OD valuation in other healthcare jurisdictions if made more consistent, providing a bridge between willingness-to-pay and budget constraint [82]. On this, a societal agreement amongst multiple stakeholders could work as a ‘collective solution for society (patients and citizens) to address rationing issues and the decision-making process’ [83]. However, while standardising decision-making processes is held as a positive step, accounting for a ‘societal perspective would not modify [pricing decisions by] healthcare payer[s], given the high ICURs identified’ because it would discount measures of ‘improvement in the state of health and well-being of other agents, such as the patients’ caregivers’ [84]. Thus, a wider set of measures appear warranted.

Here, a sub-thread holds that high(er) prices in some countries offset lower prices in poorer countries ‘by a factor of roughly 4–7 compared with uniform pricing’ [85] which serves to ‘increase the use of existing drugs (static efficiency). [in turn] increase[ing] R&D and the flow of new drugs as a result of increased sales revenue (dynamic efficiency)’ [85]. Thus, a regulatory move towards differential pricing could increase market competition while tempering OD prices over time (cf. [58]).

A second thread places an onus on market reform through regulation, often leaning towards suggestions for greater state-intervention. Regulators’ adjusting clinical and pharmacogenetic criteria could incentivise OD R&D in particular disease areas of high unmet need [81]. However, PRVs partly do this already [86], offering specific ODs faster approval and longer exclusivities. Others suggest competitive price reduction could provide ‘downward pressure to both decrease costs and improve clinical outcomes… [with] only one drug in each class [being granted].the ‘best in class’ label and demand[ing] a premium price relative to the others [87].

There are several challenges to scaling-up the above approaches, with a turn to technology proposed. Here, machine and deep learning could identifying novel side-effects and drug combinations to support better value-based pricing decisions [87], but require relevant data infrastructures, revised policy frameworks, and greater transparency of industry and regulatory valuation processes. Likewise, regulators and insurers could implement Blockchain technologies to better track actual costs [30]. Doing so within OD payment calculation could offer ‘alternative insurance mechanism[s] taking advantage of the group insurance concept’ [30]. The latter would allow patients and/or insurers to pay less while reimbursing patent holders via IPR-fees, making reimbursement processes more transparent.

In a third thread, authors suggest alternative pricing, reimbursement, and valuation processes as a path to lower OD pricing. For example, by adopting a value-added approach or ‘value informed, affordable pricing” (“VIA pricing”). in line with societal values and preferences’ [88]. Alternatively, by standardising value-added benefit assessment (notably for repurposed medicines) between countries to increase industry investment in incremental innovation [89]. Although the latter would require greater use of ‘extended evaluation frameworks and multi-criteria decision analysis (MCDA) to support priority setting, pricing, reimbursement and procurement decisions of special types of health technologies’ [89]. Others propose the EMA use Article 8.2 of Regulation EC 141/2022 to ‘accelerate [EU-wide] competition with generic products after 6 rather than 10 years of sales’ [4]. The latter, however, is contingent on resolving a pharmacoeconomic wrangle over the definition of ‘sufficient profit’. It would also require greater transparency on OD appraisal, valuation, ‘. and market access procedures… [including] current and planned indications…alternative health technologies and the total number of patients across registered and off-label indications’ [4].

Following these suggestions, some argue regulators ought to support greater use of drug repurposing and compulsory licensing, i.e. revoking patents for treatments and offering them to competitors when (bio)pharmaceutical companies profit excessively [4]. However, comparing 2007–2011 financial transaction data for OD and non-OD companies shows ‘operating profitability of orphan drug companies…[as] half that of the other companies…[with] return on equity (ROE) for investors… one-third lower than the average return observed in the other baskets’ [90], making compulsory licensing seem an unlikely solution. Moreover, returns R&D outlay fell between 1990s-mid-2010s with investors’ fearing a dip below capital costs dependent on drug price inflation [56].

To complicate matters, case law has added uncertainty over who holds remit to set OD prices. A single judge in France set the minimum price for Addmedica’s Sickle Cell Syndrome treatment *Siklos* (hydroxycarbamid), operating outside the EU regulatory framework [91]. The judge drew on American list prices as comparators rather than using the most prescribed, latest approved, and/or cheapest product. The Council of State later interceded to set an agreed price, but by then a precedent had been set. As such, the rules, devices, and boundaries between OD market and policy have been challenged, opening potential for new forms of boundary-work in shaping the market (cf. [46, 92]) - with it, a need for regulatory clarity.

In Europe, OD ICERs are also often set ‘higher than the maximum threshold for reimbursement’ [24], placing them outside usual assessment, with EU member states adopting novel price negotiation mechanisms (i.e. HST in the UK). Sensitivity and scenario analyses suggest starting negotiations earlier with alternative pricing models to reduce investor risk, thus lowering capital costs and minimum price. Applying the model to Zolgensma, for instance, a gene therapy for spinal muscular atrophy (then the world’s most expensive OD) [93] shows a single dose sits well within existing thresholds. In the Netherlands, ‘the price discount of 50%.leads to a price of €0.95 million, which is far below the lower limit of €1.7 million from the investor’s perspective.’ It does require an upfront payment of ~€2 million though, posing a risk when long-term effects are unknown, suiting a ‘staggered payment arrangement aligned with performance-based payment’ [93].

Another suggested alternative valuation approaches involves change innovation cycles. That is, because ‘pharmaceutical companies are obligated to their shareholders to increase profits’ [94], their current business and innovation models pose challenges for incentivising lower prices. Here, ‘principles of social innovation can be drawn on in the framing and articulation of such alternative pathways’ [94]– labelled social pharmaceutical innovations (SPINs). As existing SPINs, case include ‘novel R&D partnerships across the public, not-for-profit and private sectors [opening grounds for] alternative forms of provision and licensing…[with] alternative regulatory frameworks for coverage’ [94] opening potential for new pathways to lower OD costs.

A fourth thread looks holistically at the impact of high OD prices on patients’ lives in negotiating coverage/reimbursement. Within this almost 60% of rare disease patients in the USA are aged ≤ 18, giving rise to various intangible social factors, such as when ‘private insurance is tied to an employer, parents of children with rare diseases often restrict their employment choices based on the health insurance a given employer offers’ [95]. Here, alternative OD funding approaches like ‘crowdfunding [can] also make patients [and parents] heavily dependent on skills, networks, public appeal and even luck’ [96] generating inequities tied to cultural and social capital, aspects typically outside the purview of health economics. Moreover, gaining coverage for RDs often requires that parents must ‘educate insurance company representatives about their child’s disease [and/or] justify care needs’ [95]. Meanwhile, disparity between insurers’ assessment/evaluation frameworks and coverage criteria lead to unmeasured time costs and emotional strain being placed on parents and patients. If insurers employed specialists for complex care cases, while nationally ‘expanding access to Medicaid and… [shortening review times for]. applications [it] could result in additional avenues to obtain insurance [coverage]’ [95]. Moreover, it may alleviate many parents’ and patients’ concerns over how to manage the prohibitive cost of high-priced ODs. On this point, around ‘67% of US private [healthcare] insurance companies are concerned about [OD prices, yet only].17% have developed meaningful strategies for addressing [them]’ [97]. Thus, novel approaches to make coverage decisions aligned between insurers appear warranted. However, ‘regulatory justifications for providing special status to orphan drugs are ambiguous and differ between jurisdictions’ [98], raising questions about the rationale for doing so and on how insurers might address these differences within their coverage assessment processes.

In summary, literature within the third narrative weaves together various threads, with thought steeped alternatively in practical idealism and/or utilitarianism and drawing alternatively on positivist (empiricist) and more sociologically-inclined constructivist bases. They share contention that greater transparency over resource allocation decision-making is needed, balancing willingness-to-pay with budgetary constraints. That regulatory reform is needed to redress OD appraisal, valuation, and reimbursement processes. Also, that insurers could offer better assessment criteria to open new avenues for coverage while addressing intangible strain on patients. Emerging technologies and approaches to innovation are also posited as potential avenues to sustainably temper OD prices.

## Conclusion

In synthesising the three narratives (Standard 5) into a coherent meta-narrative, the review above has shown debate about OD R&D costs in the 1990s to 2000s yielding to discussion about appraisal, coverage, incentives, and reimbursement. Initially, discussion centred around the Tufts University industry-funded Center for Drug Development’s (CDD’s) controversial figure of ~$800 million (USD) for developing new OD’s. Several health economists, nonprofit organisations, and politicians in the late 1990’s claimed it as misrepresentative, with CDDs later revised (much higher) figure of $2.6 billion (USD) garnering similar rebuttals in the mid-2010s. Both drew on normative and/or empirical economics analyses read through a positivist lens, and both learned towards utilitarianism; alternatively, that both high and low OD prices best serve rare disease (RD) patients. The same underlying traditions continued throughout the mid-2000s, ranging from R&D cost calculations to consideration of treatment cost, touching on discussion of regulatory incentives, resource allocation, innovation, and market sustainability. Within this, one narrative highlighted unsavoury industry practices of drug-switching, evergreening, indication creeping, salami-slicing, the development of ‘me-too’ drugs, using ‘pay-to-delay tactics, and an emerging culture of corporate acquisitions around niche therapeutic areas. Several normative health economists hold these practices as forms of incentive misuse aimed towards excessive profiteering akin to monopoly capitalism. These were often contextualised against perceived shortfalls in OD legislation, with the FDA accused of lacking cost-effectiveness criteria during appraisal in the USA, and the EMA accused of using IP haphazardly as an incentive across Europe. However, such arguments often rely on list prices for empirical base, and a utilitarian stance where costs to/for the individual patients are foregrounded.

In rebuttal, a second narrative (pre-dominantly US-based) saw authors draw on empirical and normative economics to assert that affordability is not - and should not - be a regulatory requirement. Rather, the price of ODs produced by private companies should be left to the free-market, with high prices indicative of a healthy functioning market and likely to fall over time with increased competition. Moreover, artificially lowering prices through regulation could stall future innovation, increasing unmet need. Likewise, many note that while OD prices may seem high, further relaxation of existing cost-containment measures would better support future innovation. Thus, the narrative considers cost at a structural and societal level, drawing on a wider set of metrics to do so. The opposed positions of narratives one and two relate to a discord between expressed preference/willingness to pay and utilitarianism as underlying stances. Also, on differing positions over the appropriate level of state intervention. One proffers a rationalist notion of ODs being accessed within a free-market, the other understands that the time sensitivity of treating many RDs mean incentives such as market exclusivity provide an inelasticity to healthcare markets.

Attending to nuance within these debates, a few authors show (in a third narrative), that international referencing and differential pricing can see seemingly high OD prices occur within internal markets without fully accounting for their support of wider equities of access. As a shared contention and overarching meta-narrative, however, authors across the three narratives call for regulatory reform, both on appraisal, valuation, and reimbursement processes. Some urge greater cost-containment measures and transparency of process, others a shift in criteria and models. These discussions follow into solution-focussed arguments steeped in social science too, that looking to new models of innovation.

Whilst this review contributes to literature around orphan drug pricing, it is not without limitations. Not least in covering literature focussed on the EU, UK, and USA only. Similarly, by engaging only with items that critically engage with orphan drug pricing, the paper discounts many empirical articles and datasets that might have been drawn on to evidence or buttress critically-inclined authors’ claims - although admittedly, this article was from the outset an interpretivist examination. The article above also reviews literature over lengthy period on a topic that has evolved exceptionally quickly. New policies and approaches have emerged, with recent advances in cell and gene therapies seeing massive growth in the orphan drug market, thus many debates in older literature are likely to quickly become outdated. In policy too, the Biden administration’s (2022–2024) relatively recent enactment of the Inflation Reduction Act in 2022 saw the US begin a move towards value-based approaches to reduce drug prices– including for rare conditions [99]. However, that could be reversed by recently re-elected president Trump’s administration at any point. In short, the speed of change in both technological innovation and health economic policy surrounding rare disease treatments outpaces literature written about it. Each of these limitations provides a point of purchase for others to build on, using this paper as a background structure. Further research, for example, may examine qualitatively the cultural and social capital required for patients and/or parents to successfully negotiate access and coverage for orphan drugs. Others might model the likely impact of implementing price ceilings for ultra-rare disease treatments for orphan drug market elasticity. Likewise, a comparative article could look to review orphan drug pricing literature in other jurisdictions.

As another limitation, one key unstated finding of the review is the distinct lack of patient voice in orphan drug pricing debates literature; health economists account for a vast majority of the items, with far fewer items from activist or patient organisations. Whilst outside the bounds of this review, their conspicuous absence suggests that more ought to be done to increase and include patient input directly within critically engaged debates over OD pricing in academic and grey/policy literature. Patients and patient organisations have valuably contributed towards policy debates in the rare disease space for a long time. In the 1970’s, for instance, ACT UP successfully challenged US medical regulators via evidence-based arguments and patient stories of everyday life with HIV [100, 101], while close collaborative amongst the rare disease community led to the formation of NORD as a major umbrella organisation and locus of advocacy [102]. On orphan drug pricing in particular, protests have seen patient organisations effect direct change too. For instance, in the 1980’s, in a response to protesters hanging an effigy on New York’s Wall, drug manufacturer Burroughs Wellcome ‘slash[ed] the annual cost of treating an AIDS patient [with Retrovir] from an estimated $43,500 to $32,600’ by lowering the drug price [103]. Elsewhere, patients from at least the 2000s onwards have usurped the high costs of ODs by grouping together in ‘buyer’s clubs’ for greater bargaining power [104, 105]– made somewhat easier by social media. The latter have successfully advocated for drug repurposing (reuse of existing drug for a rare disease to reduce R&D costs too [106]. Now, patients regularly engage in meaningful community-forming dialogue with payers, regulators, and industry on a voluntary and informal basis. This is notable in the European Mechanism for Coordinated Access to Orphan Medicines (MoCA) scheme, which has informed the development and market access strategy for 23 different orphan drugs in Europe since MoCA’s 2013 founding [107]. Comparing these many contributions of patients and patent organisations, and their input towards various written outputs with a lack of presence in the search results highlights an inequity of indexing in academic databases, with grey, policy, and patient-generated items appearing less often than per-reviewed academic items– despite those databases claiming to include ‘technical information disclosed exclusively in the patent literature’ [108] alongside academic peer-reviewed texts, Scopus claiming that 36.6% of its library comprise ‘inactive journals, book series, [and] trade journals’ [109] including those without an ISSN. Here, perhaps, further work could compare this MLNR with a text analysis of patient-written literatures.

As speculative recommendations from the MLNR (Standard 6), I hold that regulators could look to emerging technologies and alternative models of innovation steeped in social science thinking when reviewing appraisal, coverage, or reimbursement processes. For example, policy-work around externally referencing prices and parallel trade agreements (where allowed) could see downward pressure reduce OD costs (and payers’ bargaining capability) in regions such as the EU. However, sensitivity would be required to avoid ‘the acceleration of access and lowering of orphan drugs net prices in high GDP EU countries while delaying or preventing access and increasing net prices in low GDP countries’ [110]– and with it a sleuth of social consequences steeped in inequity. Similarly, a move towards value-based pricing– despite the OHE’s concerns above [68]– could potentially reduce prices too, albeit with sensitivity required towards resolving various affordability challenges, including ‘drugs that meet cost-effectiveness thresholds but are “unaffordable” within the short-run budget’ of a country [111]. The latter appears to be the current direction of travel in the UK, USA and several EU member states [99, 111, 112]. Here, a prime recommendation arises, that patients should be central to any reform of OD pricing taken. Patients (including parents and carers) and patient organisations have already been actively involved with drug development– including running and inputting on innovative clinical trial designs [113]. Also as external experts with specialised expertise (e.g. therapeutic area) for joint clinical assessments and Scientific consultations during the health technology assessment of new orphan drugs [114, 115], including via organisations like MoCA [107]. However, it seems new roles could be eked out for patients to become mote meaningfully involved within priority setting and resource allocation for treating rare diseases at a structural level too, bringing diversity of lived expertise and thought to these debates.

Overall, a turn to the social sciences and focus on fostering patient agency to enact change upwards rather than expecting industry and regulators to do so structurally (downward) could see greater uptake of social innovation [94]. An existing headwind towards the latter can be seen to be emerging already as part of an increasingly mature and competitive orphan drug market ecosystem. For example, ‘[patient] advocacy foundations are adopting biomedical venture philanthropy models… [which] secures small funds for under-financed stages of therapeutic development, incentivises research, and reduces the risk inherent in novel therapy commercialisation’ [116], retaining at least some of the royalties and intellectual property rights (IPRs) for their foundation whilst lowering R&D costs for bringing new orphan drugs to market.

Partnering with universities has also lowered OD costs; the Yale University outshoot organization Universities Allied for Essential Medicines (UAEM), for example, has seen a global network of students contribute and work voluntarily towards a shared vision ‘that universities have an opportunity and a responsibility to improve global access to public health and necessary medicines’ [117] with tangible impact on pricing. In 2001, for instance, UAEM’s work ‘with Médecins Sans Frontières… [persuaded] Bristol-Myers Squibb to allow generic production of an HIV/AIDS drug in sub-Saharan Africa, resulting in a 30-fold price reduction’ [118, 119]. However, their focus has been on global equity of access rather than price per se– with little movement to lower OD costs in the UK, USA, or EU. However, elsewhere, a move towards public-private partnering with universities can brings new potential risks too. As one example, when Universities develop basic research for early-stage OD development, the research is often publicly-funded upfront and then partially reimbursed by drug sales royalties and/or through complex licence agreements with the marketing authorisation (and IPR) holding company. When public investment in public science wanes, it stands to reason that industrial partners could be left to pay more upfront for R&D to maintain innovation in their pipelines, leading to higher OD prices with less return for the university.

As a way forward, then, in working around the high OD price quandary amidst a rapidly evolving ecosystem rife with new opportunities and risks, a great deal of community-forming and compromise is now needed to adapt, with patients ideally placed to mediate discussion between industry, payers, and regulators– both to ensure access to ODs remains sustainable and to maintain market stability.

## Data Availability

A full list of reviewed literature items (including search terms) is available from searchRxiv at: 10.1079/searchRxiv.2024.00507 under a CC-BY-NC 4.0 license.

## Abbreviations

ACA: US Affordable Care Act 2010 (shortened name of PPACA)
CDD: Center for Drug Development
COMP: Committee for Orphan Medicinal Products (part of EMA)
DNDi: Drugs for Neglected Diseases initiative
EC: European Commission
EMA: European Medicines Agency
EUR: Euro (currency)
EURIPID: European Integrated Price Information Database
ERP: External Reference Pricing
FDA: US Food and Drug Administration
GBP: Great British Pound–sterling (currency)
GDP: Gross Domestic Product
HST: Highly Specialised Technology
ICER: Incremental Cost–Effectiveness Ratio
IP/IPR: Intellectual Property/Intellectual Property Rights
IRA: US Inflation Reduction Act 2022
MCDA: Multi–Criteria Decision Analysis
MHRA: Medicines and Healthcare products Regulatory Agency (UK)
MNLR: Meta–Narrative Literature Review
NHS: UK National Health Service
NICE: UK National Institute for Health and Care Excellence (UK)
OD: Orphan Drug
ODA: US Orphan Drugs Act 1983
PPACA: US Patient Protection and Affordable Care Act 2010 (also see ACA)
PPPY: Per Patient Per Year
PBM: Pharmacy Benefit Manager
PRV: Priority Review Voucher
RAMESES: Realist and Meta–narrative Evidence Syntheses: Evolving Standards
RD: Rare Disease
ROE: Return of Equity
ROI: Return on Investment
